# Thermal Hysteresis and Reversibility of the Giant Magnetocaloric Effect at the Ferromagnetic Transition of Nd_2_In

**DOI:** 10.3390/ma18133104

**Published:** 2025-07-01

**Authors:** Bao Gegen, Bao Huhe, Zhi-Qiang Ou, Francois Guillou, Hargen Yibole

**Affiliations:** 1 College of Physics and Electronic Information, Inner Mongolia Key Laboratory of Applied Condensed Matter Physics, Inner Mongolia Normal University, 81 Zhaowuda Rd, Hohhot 010022, China; 20234013021@mails.imnu.edu.cn (B.G.); 20224013014@mails.imnu.edu.cn (B.H.); zq.ou@imnu.edu.cn (Z.-Q.O.); francois.guillou@unicaen.fr (F.G.); 2 Normandie University, ENSICAEN, UNICAEN, CNRS, CRISMAT, 14000 Caen, France

**Keywords:** magnetocaloric effect, phase transitions, magnetic materials, heat capacity

## Abstract

The Nd_2_In compound exhibits an intriguing borderline first-/second-order transition at its Curie temperature. Several studies have pointed to its potential for magnetic cooling, but also raised controversies about the actual order of the transition, the amplitudes of the hysteresis, and of its magnetocaloric effect. Here, we estimate the thermal hysteresis using magnetic and thermal measurements at different rates. It is found to be particularly small (0.1–0.4 K), leading to almost fully reversible adiabatic temperature changes when comparing zero-field cooling and cyclic protocols. Some open questions remain with regard to the magnetostriction of Nd_2_In, which is presently found to be limited, in line with the absence of a thermal expansion discontinuity at the transition. The comparison of the magnetocaloric effect in Nd_2_In and Eu_2_In highlights that the limited saturation magnetization of the former affects its performance. Further efforts should therefore be made to design materials with such borderline first-/second-order transitions using heavier rare earths.

## 1. Introduction

Rare-earth indium *R*_2_In compounds form a relatively large materials family with intriguing magnetic properties; members of this family have recently attracted researchers’ interest due to their potential magnetocaloric applications. *R*_2_In compounds based on trivalent rare earths crystallize in a hexagonal Ni_2_In-type crystal structure (space group P6_3_***/****mmc*) featuring two inequivalent rare earth sites with distinct coordination geometries [1]. Meanwhile divalent-like Eu_2_In and Yb_2_In compounds crystallize in a Co_2_Si-type orthorhombic structure (space group P*nma*), also comprising inequivalent sites [2,3]. Most of the hexagonal *R*_2_In with *R* = Nd, Pr, Gd, Tb, Dy, Ho, Er, Tm exhibit ferromagnetism with Curie temperatures loosely following a De Gennes behavior [4,5]. Yet, several compounds from the series present unique features that are of particular interest. Sm_2_In shows ferrimagnetic ordering with small spontaneous magnetization [6]. Gd_2_In actually presents an antiferromagnetic ground state turning into ferromagnetism through a metamagnetic transition at about 100 K [7]. The metamagnetic transition of Gd_2_In is remarkable. It is of the first-order type and can be induced by temperature, magnetic field, or pressure, but is associated with a limited latent heat and hysteresis [8,9,10,11,12]. More recently, the search for materials for magnetic cooling and the observation of a giant magnetocaloric effect in Eu_2_In involving a rather unique mechanism has triggered a renewed interest for *R*_2_In compounds [13,14,15]. Whereas the heavy rare earth shows continuous second-order ferromagnetic transitions, the light rare-earth Nd_2_In and Pr_2_In present first-order ferromagnetic transitions (FOMT) [16,17,18,19]. The latent heat of these FOMTs leads to isothermal entropy change (Δ*S*) and adiabatic temperature change (Δ*T*_ad_) larger than that observed for the heavy rare earth *R*_2_In despite the lower saturation magnetization of the light rare earths [20,21,22]. In combination with a broader availability of light rare earths, it makes Pr_2_In and Nd_2_In potential magnetocaloric materials for cryogenic cooling with performances on par or greater than those of other magnetocaloric material families [23,24,25,26,27,28,29], especially when looking at the temperature range relevant for the liquefaction of natural gases.

Unlike conventional FOMT materials such as Gd_5_Si_2_Ge_2_, MnAs, FeRh, La(Fe,Si)_13_ or MnFe(P,As) which exhibit crystal symmetry breaking, large volume changes or pronounced lattice distortions [30], Nd_2_In was found to be nearly structurally invariant across its magnetic transition, with a temperature-dependent crystallographic study revealing no symmetry breaking or discontinuities in lattice parameters [19]. Its ferromagnetic transition therefore appears to be on the borderline between the first- and second-order type. As a result, while three nearly simultaneous studies have confirmed the potential of Nd_2_In for use in magnetic cooling [17,18,19], the exact nature of the ferromagnetic transition remains subject to debate. Using the same criteria—for instance, the shape of the heat capacity peak or the field dependence of the isothermal entropy change—the transition has been alternatively suggested to be of first- or second- order. The report of large magnetostrictive strains at the transition also contrasts with the nearly negligible volume change from temperature-dependent diffraction experiments. In addition, the thermal hysteresis of the transition shows a scatter with illustrations presenting an inverted hysteresis [17], with reports mentioning almost no thermal hysteresis or hysteresis less than 2 K [18,19]. Finally, the amplitude of the magnetocaloric effect itself presents some discrepancies, with adiabatic temperature changes ∆*T*_ad_ ranging from about 1.8 K for µ_0_∆*H* = 2 T from indirect heat capacity measurements down to 1.13 K for µ_0_∆*H* = 1.95 T from direct cyclic measurements.

Since only first-order transitions can present a finite thermal hysteresis, a relatively accurate determination of the hysteresis would be needed to settle the controversies on the nature of the phase transition. The question of the hysteresis is also essential for assessing magnetocaloric performances. If thermal hysteresis is present, it would affect the reversibility of the phase transition upon magnetization/demagnetization cycles and therefore would lead to a decrease or even an absence of magnetocaloric effect after the first field application. One can actually note that a finite hysteresis could be the origin of the differences in the reported ∆*T*_ad_ values, since hysteresis would affect only direct cyclic measurements. Here, we revisit the magnetocaloric effect and the ferromagnetic transition in Nd_2_In by using thermal measurements. We pay special attention while separating the dynamic contributions that may hinder the estimate of the thermal hysteresis during magnetic or thermal properties measurements. We observe a particularly small, yet finite, thermal hysteresis of 0.1–0.4 K. This hysteresis is found to be sufficiently limited to not result in noticeable losses during cyclic ∆*T*_ad_ measurements. Finally, a descriptive parametric model is applied to shed light on the origin of the ∆*T*_ad_ difference between Eu_2_In and Nd_2_In.

## 2. Materials and Methods

**Figure 1:**
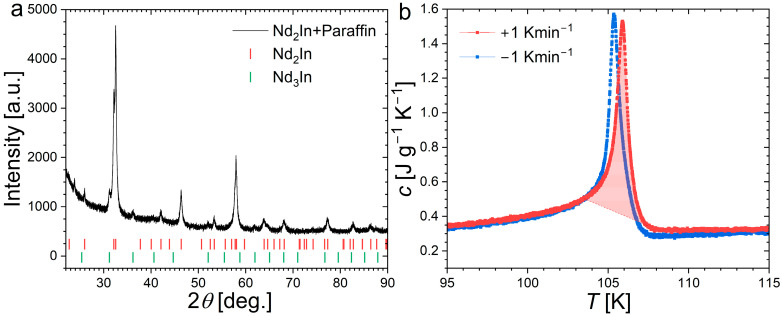
Panel (**a**): Powder X-ray diffraction pattern measured at room temperature on Nd_2_In powder dispersed in paraffin oil. The ticks mark out the reflections corresponding to Nd_2_In and Nd_3_In structures. Panel (**b**): specific heat of Nd_2_In recorded by a Peltier Cell Differential Scanning Calorimeter upon heating and cooling. The highlighted area corresponds to the integration of the transition entropy change.

A polycrystalline Nd_2_In sample was prepared by arc melting followed by heat treatment. The elemental starting materials were arc-melted 4 times in a purified Ar atmosphere while flipping the button at each iteration. The resulting button was sealed into a quartz ampule and annealed at 700 °C for 100 h. Powder X-ray diffraction measurements were carried out at room temperature using a PANalytical Empyrean diffractometer (Malvern Panalytical, Malvern, UK). The powder was crushed and sieved in an Ar-purified glovebox, then mixed with paraffin oil to prevent oxidation during the XRD measurement. Figure 1 shows the resulting diffractogram, which can be indexed and refined using the Rietveld method in the reported hexagonal structure with lattice parameters *a* = 5.5002(6) Å and *c* = 6.8684(9) Å, consistent with former reports [1,19,31]. In addition to a significant background present at low angle due to the paraffin oil, additional peaks are visible at 31.2° and 36.2°, which can be attributed to a cubic Nd_3_In secondary phase (6.6(4)wt.%). A similar Nd_3_In secondary phase was also observed in previous reports [18,19].

Physical properties were recorded using a Quantum Design Versalab cryostat (Quantum Design, San Diego, CA, USA). Magnetization measurements were carried out using a Vibrating Sample Magnetometry option. A homemade thermometry option close to the principle of a Differential Thermal Analyzer was used to record thermograms across the phase transition. This DTA was built by fixing two thermal sensors (one for sample, one for reference) at the extremities of two conic pillars made of PLA on a blank Versalab puck. Sample and reference temperatures were read by using bare chip Cernox sensors (CX-1070-BC-HT, Lake Shore Cryotronics, OH, USA) with a fast response time and limited thermal dilution (sensor mass << sample mass). The sensor resistivity yielding the temperature after calibration was recorded using a built-in Quantum Design Electrical Transport Option, with the same excitation amplitude (1 mA) and different frequencies (15.26 and 21.36 Hz, respectively) for sample and reference channels. Similar sensors and electronics, mounted on a different sample holder, were found to be effective in recording thermal events associated with FOMT. This included capturing fine burst-like features that developed during the transition, as observed in La(Fe,Co,Si)_13_ giant magnetocaloric materials [32].

Direct ∆*T*_ad_ measurements were carried out using the DTA hardware by sweeping the magnetic field at a rate of µ_0_d*H*/d*t* = 1.8 Tmin^−1^ in high-vacuum conditions. For ∆*T*_ad_ measurements at µ_0_∆*H* = 2 T, the initial temperature before field application was reached after a zero-field cooling from the paramagnetic state, and two successive magnetization/demagnetization cycles were recorded. For ∆*T*_ad_ (µ_0_∆*H* = 1 T), only cyclic Δ*T*_ad_ were recorded (no ZFC). The sample for ∆*T*_ad_ measurement is relatively large (1.101 g) and the thermal exchanges are limited, so that appropriate adiabatic conditions are ensured during the magnetic field change. If a single field application is applied, the subsequent exponential thermal relaxation of ∆*T*_ad_ to the initial temperature shows a typical relaxation time constant of ~30 min, which is considerably longer than the actual ∆*T*_ad_ measurement time. The robustness of this Δ*T*_ad_ setup without extraction was confirmed by measuring a Gd reference sample. The resulting ∆*T*_ad_ = 3.06 K for µ_0_∆*H* = 1 T was found to be slightly lower, yet within 4% of direct measurements using rotative magnetic field with µ_0_d*H*/d*t* ≈ 1 Ts^−1^ on Gd samples of similar purity [33].

Heat capacity measurements were carried out using a Quantum Design heat capacity option equipped with a vertical puck kit. The built-in 2τ analysis is used for measurements between 50 and 200 K and complemented by Single-Pulse Measurements (SPMs) near the Curie temperature [34]. Since Differential Scanning Calorimetry is often considered as the most suitable technique for recording the latent heat of FOMT, a complementary DSc measurement was carried out on Nd_2_In. Figure 1 presents the measurements carried out near the FOMT using a homemade Peltier cell DSC for a Quantum Design cryostat [32]. The integration of the *c*/*T* peak subtracted by a linear background yields a transition entropy change of 10.9 J kg^−1^ K^−1^.

Dilatometry measurements were carried out using a homemade strain gauge option. ZEMIC BAB-120-3AA250(11) (Xi’an, China) strain gauges were bonded with H-610 epoxy on a polished 9 × 9 × 5 mm^3^ bulk piece of Nd_2_In and a fused silica reference sample. A Keithley 2400 source meter unit was used for the voltage excitation of the Wheatstone bridge and a Keithley 2182A nanovoltmeter was employed to record the output voltage signal; both were controlled via a ^2018^LabVIEW interface (Tektronix, OR, USA). Prior to the measurement, a Cu metal reference (Puratronic, 99.999%, Alfa Aesar, MA, USA) was measured by using gauges originating from the same batch (and the same glue). At *T* = 293 K, the measured linear thermal expansion for the cupper, 16.8 ppmK^−1^, was in line with the expected value and over the whole temperature range (50–350 K), deviations from the NIST reference values did not exceed 2.5%.

## 3. Results

**Figure 2:**
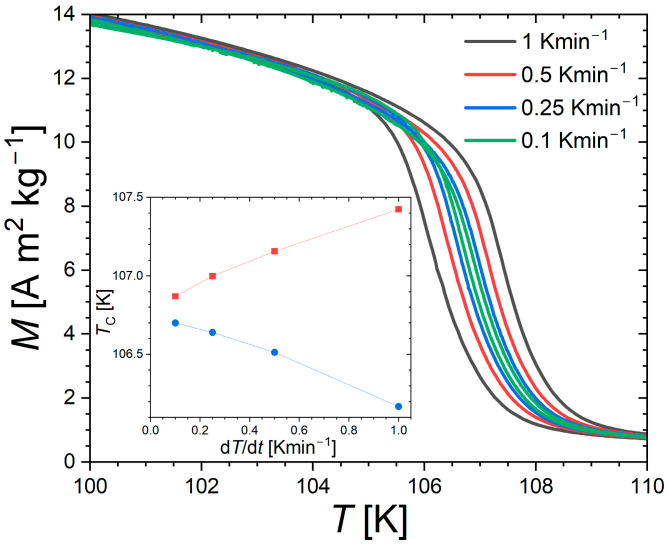
Temperature dependence of the magnetization in µ_0_*H* = 0.1 T recorded at different sweeping rates upon heating and cooling. In the inset, transition temperatures are shown upon heating (squares) and cooling (circles). The transition temperatures were determined as the maxima on derivative |d*M*/d*T*| curves.

Figure 2 presents magnetization measurements as a function of the temperature near the Curie temperature of Nd_2_In. In line with former reports [4,5,17,18,19], a relatively sharp drop in magnetization is observed at about 108 K. As the present study is devoted to the main ferromagnetic transition in Nd_2_In, the comparatively minor magnetic anomaly usually observed at lower temperature is not investigated (spin reorientation transition near ~50 K) [17,18,19]. At the ferro–paramagnetic transition, a finite thermal hysteresis is observed between measurement upon heating and cooling. However, this dataset, recorded at different sweeping rates, successfully illustrates the difficulty of separating the intrinsic thermal hysteresis of the phase transition from the thermal lag inherent to dynamic measurements. At the lowest rate (0.1 Kmin^−1^), a finite thermal hysteresis δ*T*_hyst_ ≈ 0.18 K is observed. This value turns out comparable with the exceptionally small hysteresis δ*T*_hyst_ ≈ 0.1 K at the first-order ferromagnetic transition of Eu_2_In [13] and one order of magnitude smaller than typically observed in archetypical giant magnetocaloric materials with optimized hysteresis, such as La(Fe,Si)_13_ or MnFe(P,Si) [35,36,37].

**Figure 3:**
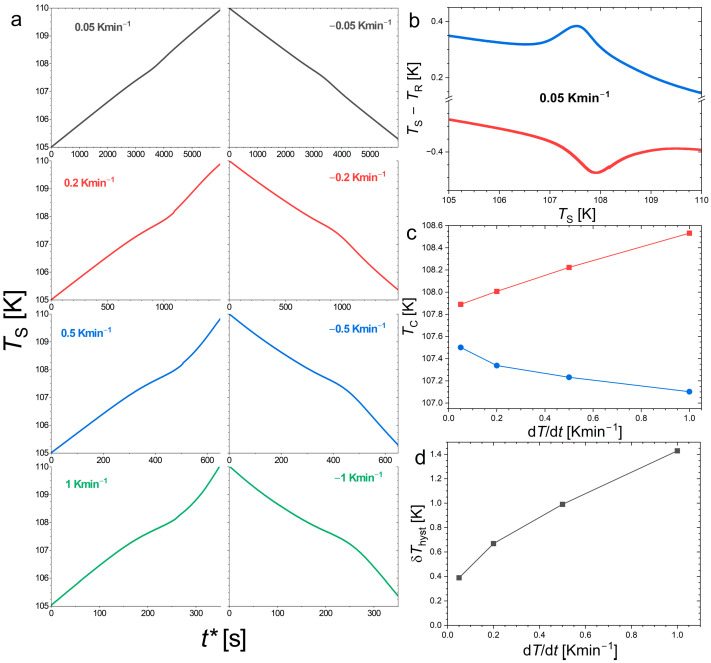
Thermal measurements carried out at different sweeping rates at the ferromagnetic transition of Nd_2_In. (**a**) Sample temperature (*T*_S_) thermograms recorded in DTA upon heating (**left**) and cooling (**right**), with the time axis presented as t* = *t* − *t* (*T* = 105 K) and t* = *t* − *t* (*T* = 110 K), respectively. (**b**) DTA signal at the slowest sweeping rate of ±0.05 Kmin^−1^ upon heating (**bottom**) and cooling (**top**). (**c**) Transition temperature upon heating (squares) and cooling (circles) determined as d*T*/d*t* minima on the thermograms. (**d**) Thermal hysteresis determined from the difference in transition temperatures.

Unfortunately, magnetic data achieved with finer control of the temperature—for instance, by stabilizing the temperature at each temperature point—would not yield a more accurate estimate of the hysteresis. Typically, during the thermalization to a new temperature, the cryostat stabilizes through decaying thermal oscillations around the target temperature. If the amplitude of these oscillations is on par with the thermal hysteresis, the thermomagnetic history of the sample can no longer be tracked. Different measurement protocols, particularly different sweeping rates, are likely responsible for the scatter in the reported thermal hysteresis values for Nd_2_In [17,18,19]. To obtain a more reliable estimate of δ*T*_hyst_, we turned to methods which involved taking a direct reading of the actual sample temperature during the measurements. For instance, DTA is one of the most traditional methods for detecting phase transitions. Figure 3 presents DTA thermograms at different sweeping rates in Nd_2_In. A thermal anomaly marks out the ferromagnetic transition near 108 K, but it corresponds to a relatively broad feature, without a clear plateau of constant temperature expected due to the latent heat of a first-order transition. Yet, the DTA vs. T curves still correspond to a notable peak. Even if the thermal sensor is connected (by using grease) to the sample, measurements at different rates allow a progressive dynamic contribution to appear. The smallest measured hysteresis is δ*T*_hyst_ ≈ 0.38 K, which extrapolates to the range 0.4–0.2 K for static conditions.

If DTA is well suited to detecting phase transitions while offering a broad dynamical range, it provides only a limited accuracy for quantitative analysis. Specific heat measurements were therefore carried out by using the standard analysis of the heat capacity option of a Quantum Design cryostat from 50 to 200 K in various magnetic fields and complemented by the Single-Pulse Method (SPM) near the Curie temperature. SPM involves applying large heat pulses to fully cross the transition, and it offers a better temperature incrementation, allows the separation of heating and cooling branches, and allow us to overcome the limitations of the built-in 2τ method [34,38,39,40].

**Figure 4:**
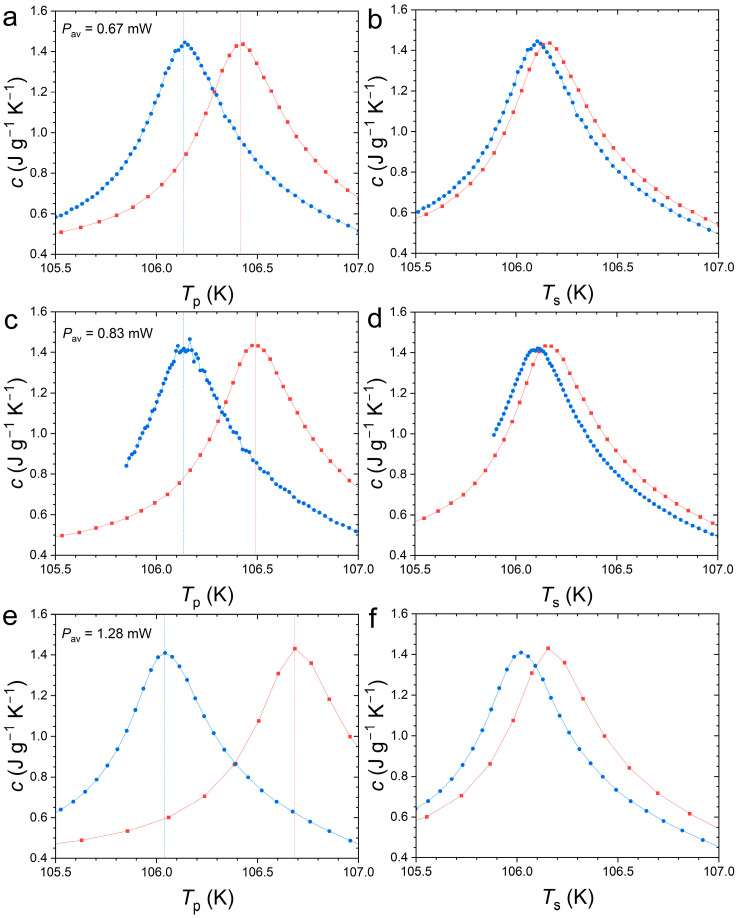
Specific heat for Nd_2_In in *H* = 0 determined by the Single-Pulse Method using different heating powers upon heating (squares) and cooling (circles). (**a**,**c**,**e**), raw data presented as a function of the platform temperature (*T*_p_); (**b**,**d**,**f**) corresponding data corrected for thermal lags (as a function of the sample temperature (*T*_S_)).

Figure 4 presents the specific heat peak measured by SPM at the Curie temperature of Nd_2_In. First, one can note that the thermal anomaly associated with the FOMT is relatively successfully captured by the SPM method, as it yields a peak maximum of ~1.5 J g^−1^ K^−1^ comparable to DSC measurements presented in Figure 1. We also observe that the peak marking the transition is rather symmetric. A Gaussian distribution of latent heat centered on the transition temperature is typical of first-order transitions and contrasts with the lambda-like anomaly of second-order transitions. The improved temperature resolution offered by SPM allows us to accurately describe the specific heat event, which may explain the difference compared to the original report on Nd_2_In concluding on a lambda-like anomaly [15]. When analyzed in a 1τ model considering the sample and platform as a single system in perfect thermal contact, the specific heat is expressed as a function of the platform temperature (*T*_P_). By selecting a different bath temperature (*T*_0_) and targeted pulse temperature range, different amounts of heat power can be applied. Increased heat power leads to a faster crossing of the transition and also results in an apparent increase in thermal hysteresis between the peak upon heating and cooling as a function of *T*_P_, corresponding to δ*T*_hyst_ in the range 0.29 to 0.64 K for the explored SPM parameters.

**Figure 5:**
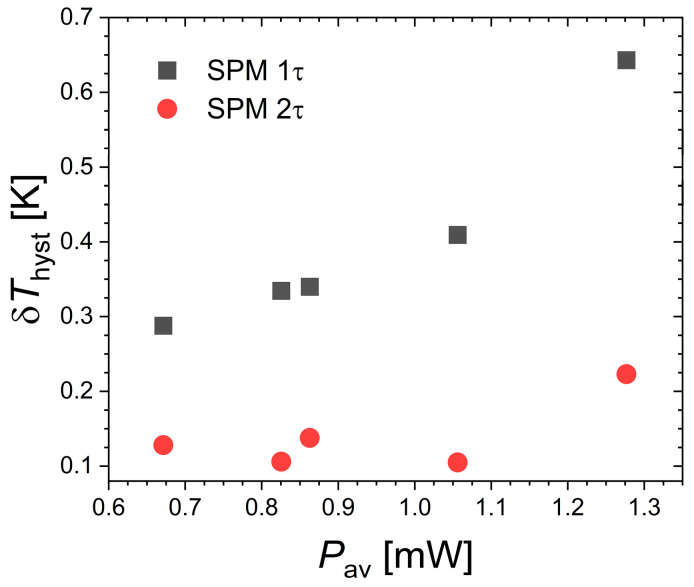
Thermal hysteresis at the ferromagnetic transition of Nd_2_In determined by specific heat (SPM) in *H* = 0 within 1τ and 2τ thermal models.

The increase in δ*T*_hyst_ with the applied heat again illustrates the difficulty of estimating the thermal hysteresis even in experimental setups in which the thermometer is in contact with the sample. To take into account the thermal lag between sample (*T*_S_) and platform (*T*_P_) temperatures, one has to involve a second heat balance which accounts for a finite conductance of the grease [34,38,39,40]. The thermal lag $T_{S}-T_{P}=\left[c_{add}\frac{dT_{P}}{dt}-P+K_{w}\left(T_{P}-T_{0}\right)\right]/K_{g}$ can be estimated using the grease *K_g_* and wire *K_w_* conductance determined by extrapolation of the 2*τ* fittings before and after the transition. Figure 4 illustrates the difference between the raw data as a function *T*_P_ and the corrected data vs. *T*_S_ and Figure 5 summarizes δ*T*_hys_ from the peak maxima for the different pulses. Correcting for thermal lags leads to a reduced, yet non-vanishing, hysteresis of 0.1 to 0.2 K. However, the thermal lag correction, and therefore hysteresis, is highly sensitive to the estimation of *K_g_*, which is a parameter that is indirectly estimated. Unfortunately, independently measuring or calibrating *K_g_* is challenging as it depends on the amount of grease and the sample positioning, both of which are difficult to reproduce between different measurements. It should be noted here that a reduction in *K_g_* by 40% would be required to cancel out the hysteresis, which at first glance seems rather unlikely.

**Figure 6:**
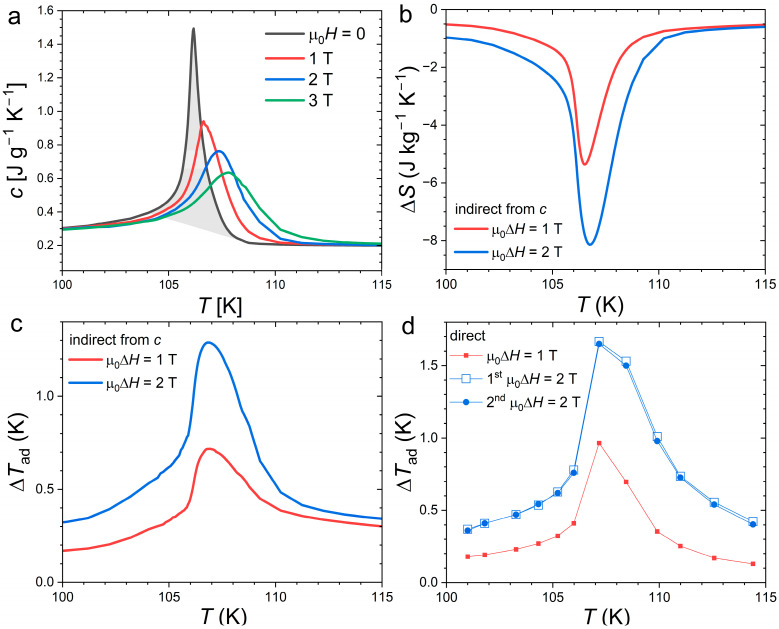
Specific heat and magnetocaloric effect of Nd_2_In. (**a**) Specific heat as a function of the temperature in zero and for different magnetic fields (built-in 2τ analysis outside the transition complemented by SPM at *T*_C_). The shaded area illustrates the transition entropy (Δ*S*_tr_). (**b**) Isothermal entropy changes (Δ*S*) and (**c**) adiabatic temperature changes (Δ*T*_ad_) indirectly calculated from specific heat data. (**d**) Δ*T*_ad_ from direct measurements. For 2 T, the first measurement after a zero-field cooling and subsequent cycle are presented.

Figure 6 presents measurements of the specific heat in applied magnetic fields and the corresponding determination of the magnetocaloric effect as well as a comparison with direct Δ*T*_ad_ measurements. The transition entropy change was estimated by integration of the specific heat using a linear background yielding a value $∆S_{tr}=\int_{104\;K}^{108\;K}\frac{c-c_{back}}{T}dT\approx \;$10.8 J kg^−1^ K^−1^. A comparable estimate (10.9 J kg^−1^ K^−1^) was obtained using DSC data, as shown in Figure 1, which confirms the reliability of the present specific heat data even at the FOMT. In addition to measurement techniques, estimating the transition entropy is actually made tricky by the selection of the starting and end points of the transition. Here, even when considering a temperature window of 4 K, which is relatively large with regard to the ~0.7 K full width at half maximum of the peak, the Δ*S*_tr_ in Nd_2_In appears thrice smaller than that found in orthorhombic Eu_2_In (Δ*S*_tr_ ≈ 27.5 J kg^−1^ K^−1^ [13]) indicating a significantly weaker FOMT.

The application of a magnetic field yields a pronounced broadening of the specific heat peak, which highlights the relatively weak character of the FOMT in comparison to strong latent heat system for which the peak intensity is more robust upon high field application. The shift in the transition due to the field estimated from the specific heat maxima in different fields is linear and yields a relatively modest $\frac{dT_{tr}}{\mu_{0}dH}=+0.6\;\left(1\right)\;KT^{-1}$. The isothermal entropy change indirectly derived from calorimetry data corresponds to a maximum of −8.2 J kg^−1^ K^−1^ for µ_0_Δ*H* = 2 T, well within the range of previously reported values (from −7.42 to −13 J kg^−1^ K^−1^ [17,18,19]). The adiabatic temperature change was estimated by indirect specific heat measurements at 1.3 K for 2 T. Similarly to Δ*S*, the Δ*T*_ad_ performances are found to be intermediate among the previously reported results [17,18,19].

More specific to our study, we performed direct ∆*T*_ad_ measurements at the first magnetization after zero field cooling and upon further demagnetizing/magnetization cycles. The differences between the first and second magnetizing measurements turned out to be insignificant (differences of 0.03 K or less), demonstrating that the MCE is nearly fully reversible upon cycling, even at an intermediate applied magnetic field of 2 T. The Δ*T*_ad_ from direct measurement reaches 1.68 K for 2 T, which is apparently larger than that found from indirect measurements. The difference may originate from the uncertainty of each technique. In particular, the indirect method suffers from several possible sources of error, such as underestimating the latent heat, cumulative errors during the entropy integration, and starting the integration from a temperature different from 0 [41,42].

**Figure 7:**
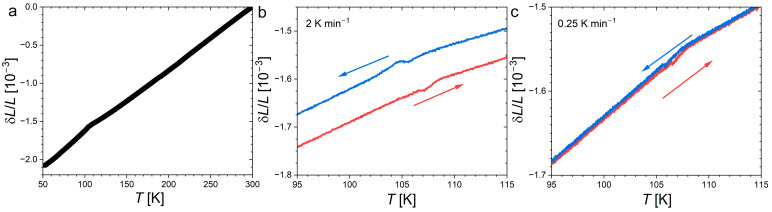
(**a**) Linear thermal expansion of Nd_2_In in *H* = 0. Enlargement on the ferromagnetic transition; (**b**,**c**) measurements at 2 Kmin^−1^ and 0.25 Kmin^−1^, respectively. The arrows indicate the heating/cooling directions.

**Figure 8:**
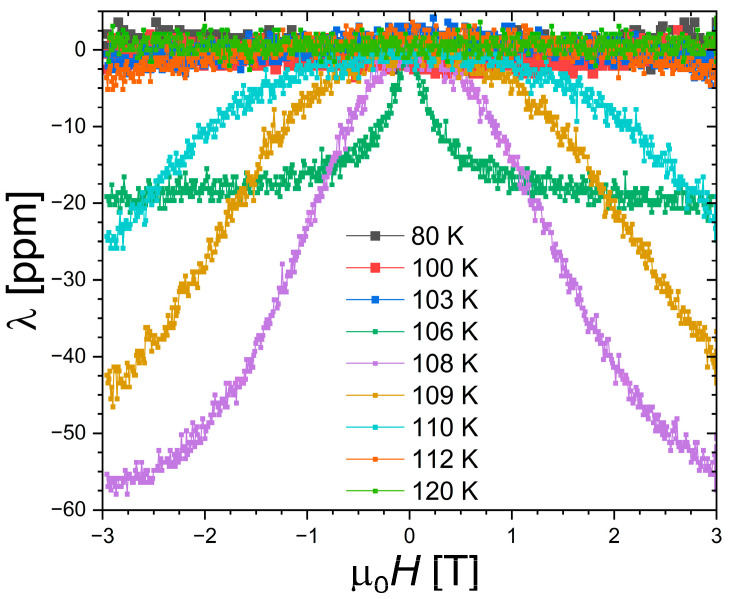
Longitudinal magnetostriction for Nd_2_In measured at different temperatures.

Thermal expansion and magnetostriction of Nd_2_In are presented in Figure 7 and Figure 8, respectively. In line with former dilatometry or temperature-dependent XRD experiments [17,18,19], the ferromagnetic transition does not correspond to a significant discontinuity, but is fingerprinted by the appearance of a negative spontaneous magnetostriction. The absence of marked anomaly contrasts with the large volume or cell parameter changes usually observed at the FOMT of giant MCE materials. In the paramagnetic state, above 150 K, the thermal expansion α_L_ ≈ 8.5 ppmK^−1^ is rather linear and is in line with that observed from powder XRD, α_V_ ≈ 28 ppmK^−1^ [19]. Below *T*_C_, the linear expansion is larger and amounts to α_L_ ≈ 9.8 ppmK^−1^.

A close inspection of the thermal expansion near the ferromagnetic transition reveals a small bump and a finite thermal hysteresis. Both features are strongly suppressed by reducing the sweeping rate, suggesting they mainly originate from sample thermalization. At the slowest rate of 0.25 Kmin^−1^, a thermal hysteresis of δ*T*_hyst_ ≈ 0.4 K is observed.

Longitudinal magnetostriction measurements were carried out at selected temperatures, below, at, and above the ferromagnetic transition. In the ferromagnetic state, the forced magnetostriction is small (≈2 ppm for 3 T at 80 K). A finite negative longitudinal magnetostriction appears near the phase transition, with an S-shape just above *T*_C_, mimicking the magnetization of a field-induced FOMTs. This magnetostriction quickly vanishes when the temperature is raised (or requires significantly larger magnetic field to be induced), and become negligible (≤1 ppm) in the paramagnetic state above 120 K. Qualitatively, the presently observed temperature dependence of the magnetostriction is in overall agreement with the former two reports [17,18]. Quantitatively, however, one can note some notable differences. In ref. [18] employing strain measurements as a function of the temperature in different magnetic fields, a negative magnetostriction was observed only in applied fields of 8 T and more. Here, finite negative magnetostriction is observed at intermediate fields of 1 or 2 T during isothermal measurements. Differences in thermalization might influence the observation of subtle magnetostrictive features. With a progressive saturation just above *T*_C_ at −58 ppm for 3 T, the amplitude presently observed is significantly smaller than the giant saturation magnetostriction of ~450 ppm reported in ref. [17].

## 4. Discussion

As pointed out by Biswas et al. [19], the classification of the Nd_2_In ferromagnetic transition into first- or second-order transition has turned out to be tricky. The absence of noticeable discontinuity on the thermal expansion suggests a continuous transition, whereas the rather symmetric heat capacity peak is more typical of a FOMT. In this context, the presence of a thermal hysteresis distinctive of FOMT is important. But, as illustrated here, hysteresis is also particularly challenging to measure with a reasonable accuracy, which is the primary reason for the scatter among the reported values for Nd_2_In. Magnetization, DTA, specific heat, and dilatometry performed at different rates suggest a finite δ*T*_hyst_, but of exceptionally small amplitude of 0.1 to 0.4 K, comparable to that observed in Eu_2_In [13]. While Nd_2_In definitively lays at the first-/second-order boundary, the persisting finite hysteresis suggests this ferromagnetic transition is more likely to be a FOMT.

Giant saturation magnetostriction [17] or field-induced transition processing in two stages on strains [18] were not observed. Some aspects of the magnetostriction of Nd_2_In therefore remain elusive and would require further investigation. One can, however, point out that the absence of thermal expansion anomaly at the transition is, at first glance, in line with the modest longitudinal magnetostriction presently observed at *T*_C_.

**Figure 9:**
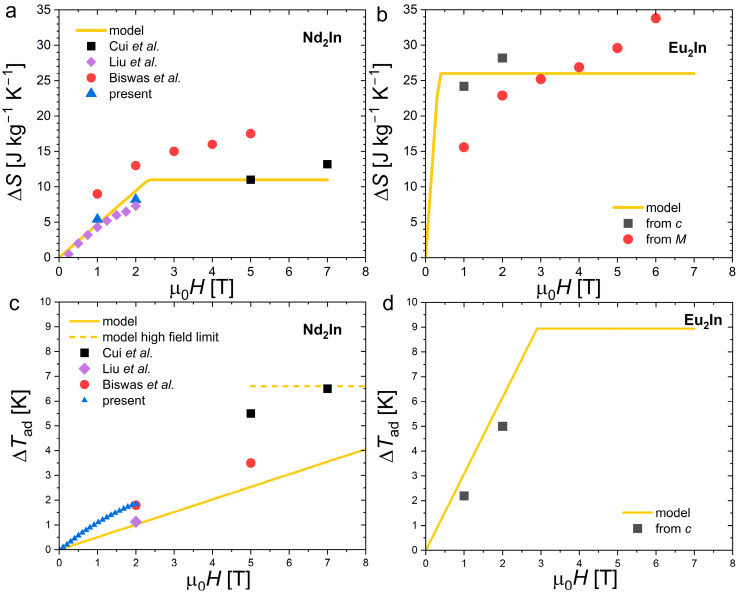
Comparison of magnetocaloric performances between Nd_2_In and Eu_2_In. (**a**,**b**) Isothermal entropy change (Δ*S*) for Nd_2_In and Eu_2_In, respectively. (**c**,**d**) Adiabatic temperature change (Δ*T*_ad_) for Nd_2_In and Eu_2_In, respectively. Refs. [17,18,19] are cited in the figure.

The very limited thermal hysteresis leads to nearly identical Δ*T*_ad_ between measurements after a ZFC and those measured in cyclic conditions. The scatter in reported Δ*T*_ad_ values for Nd_2_In does not, therefore, originate from irreversibility. It most likely involves the uncertainty inherent to the Δ*T*_ad_ measurement methods and the sample preparation; in particular, parameters influencing the sharpness of the transition such as the raw materials’ purity or the sintering conditions. However, no direct relation can be established between phase purity and magnetocaloric performances, as Δ*S* or Δ*T*_ad_ do not appear to scale with the Nd_3_In weight fractions in the various studies. For instance, our sample appears to suffer from the largest Nd_3_In contamination, but the presently observed Δ*T*_ad_ ≈ 1.7 K for 2 T are intermediate values among the different reports on Nd_2_In [17,18,19]. The magnetocaloric effect of Nd_2_In is significant compared to other materials in this temperature range, but it remains notably smaller than Δ*T*_ad_ ≈ 5.0 K for 2 T found in the closely related Eu_2_In [13]. Figure 9 summarizes the experimental Δ*S* and Δ*T*_ad_ data available for both compounds in different magnetic fields, and a parametric latent heat model used for their interpretation.

Among the various approaches which can be used to predict the magnetocaloric effect, a parametric “latent heat” method can appropriately describe the giant magnetocaloric effect with FOMT. This model consists of describing the shift in the entropy discontinuity of a FOMT due to the application of a magnetic field [43,44,45,46,47,48]. At high magnetic fields, when the transition is fully induced, Δ*S* corresponds to the entropy change in the transition Δ*S* = Δ*S*_tr_ and correspondingly Δ*T*_ad_ = *T*_C_Δ*S*_tr_/*c*_back_, where *c*_back_ is the specific heat background outside the transition. Below their respective saturating fields, Δ*S* = Δ*S*_tr_[Δ*H*(d*T*_tr_/d*H*)]/δ*T*_tr_ and Δ*T*_ad_ = Δ*H*(d*T*_tr_/d*H*)[1 − δ*T*_tr_/(Δ*S*_tr_/*c*_back_ + δ*T*_tr_)] are dependent on the magnetic field change (Δ*H*) and can be expressed as a function of several externally measured parameters such as the shift in the transition due to field (d*T*_tr_/µ_0_d*H*) or the width of the transition (δ*T*_tr_) [43,44,45,46,47,48]. While it is advantageous to describe giant MCE*s*, this model neglects the magnetocaloric contributions that do not originate from the FOMT (the magnetocaloric effect due to application of the field on the paramagnetic and ferromagnetic fractions below and above the FOMT). Table 1 summarizes the parameters used to model Nd_2_In and Eu_2_In in Figure 9.

Applying the model to the case of Nd_2_In and Eu_2_In yields only a modest agreement with the experimental results. But the apparent deviation also originates from the large scatter on the experimental values. One is thus limited to a discussion of the overall tendencies which remain relatively well captured by the model. Regarding Δ*S*, the larger Δ*S*_tr_ for Eu_2_In than Nd_2_In favors significantly larger Δ*S* at high fields. In addition, large Δ*S* values are reached sooner in Eu_2_In than Nd_2_In at intermediate fields (1 T). While both compounds present very sharp transitions, the difference primarily originates from the greater sensitivity to the magnetic field in Eu_2_In (d*T*_tr_/µ_0_d*H*), which is nearly six times larger than that presently observed in Nd_2_In. The Clausius Clapeyron relation d*T*_tr_/µ_0_d*H =* −Δ*M*/Δ*S*_tr_ implies that this larger d*T*_tr_/µ_0_d*H* mostly originates from the larger magnetization change at the transition. Δ*M* is a parameter involving extrinsic aspects such as the sharpness of the transition, but it is also bounded by the saturation magnetization which is primarily attributed to the nature of the rare earth in these compounds. With g*J* = 7 μ_B_ for Eu^2+^ in comparison to 3.27 μ_B_ for Nd^3+^, Eu_2_In shows a twice larger saturation magnetization and an even greater Δ*M* than Nd_2_In due to its more pronounced FOMT characteristics.

Noting that the specific heat outside the transition *c*_back_ is relatively high for both Nd_2_In and Eu_2_In, it negatively affects the Δ*T*_ad_ to a similar extent. Greater d*T*_tr_/µ_0_d*H* also favors larger Δ*T*_ad_ in Eu_2_In at intermediate fields. High field Δ*T*_ad_ measurements on Eu_2_In would, however, be needed to expand the comparison.

## 5. Conclusions

Magnetization and thermal measurements carried out in different conditions indicate an exceptionally small thermal hysteresis of ~0.1 to ~0.4 K at the first-order ferromagnetic transition of Nd_2_In. Adiabatic temperature changes measured after resetting the thermomagnetic history and in cyclic conditions show that the hysteresis does not lead to significant losses upon cycling, further confirming the interest of this compound for MCE applications. A limited longitudinal magnetostriction is observed at the field-induced transition, which is in line with the absence of a significant anomaly on the thermal expansion as a function of the temperature. The comparison of the MCE between Nd_2_In and Eu_2_In highlights that a large amount of magnetization remains one of the most important parameters for achieving exceptional performances. Further research on *R*_2_In materials should therefore focus on combining the isostructural FOMT transition observed in light Nd_2_In, Pr_2_In and Eu_2_In compounds with the larger moments offered by heavy rare earths, such as those possibly occurring in Er_2_(In,Al) [49]. Additionally, the underlying mechanism responsible for the nearly non-hysteretic FOMT in *R*_2_In compounds warrants further investigation, particularly with regard to its generalization across various rare earths.

## Figures and Tables

**Table 1:** Parameters used to model the magnetocaloric effect of Nd_2_In and Eu_2_In.

Compd.	*T*_C_	Δ*S*_tr_	d*T*_tr_/µ_0_d*H*	*c*_back_	δ*T*_tr_
Units	K	J kg^−1^ K^−1^	K T^−1^	J kg^−1^ K^−1^	K
Nd_2_In	108	10.8	0.6	180	1.2
Eu_2_In ^1^	55	27.5	3.5	160	1.2

^1^ Ref. [13].

## Data Availability

The original contributions presented in this study are included in the article. Further inquiries can be directed to the corresponding author.
